# The Government Meat Inspection*Office of Information, U. S. Department of Agriculture, Washington, D. C.

**Published:** 1913-10

**Authors:** 


### The Government Meat Inspection.*

The magnitude of the Government Meat Inspection Service is shown by the following figures covering the past seven years, the period during which the present law has been in effect. In this period more than 377,000,000 animals were inspected at slaughter, of which 1,100,00 carcasses and 4,750,000 parts of carcasses were condemned. The reinspection of meat and meat food products in their various preparations amounted to 44 billion pounds, of which there were condemned on reinspection 148,000,000 pounds. There were certified for export 8 billion pounds.

Federal inspection is maintained at 792 slaughtering and packing establishments, which number includes practically every establishment of importance in the country. These establishments are distributed in 227 towns and cities. The force necessary to conduct inspection is comprised of 2,400 veterinary inspectors and assistants.

The quickness and certainty with which the government inspectors stationed in packing houses detect tuberculosis and other diseases in animals and carcasses examined by them at the time of slaughter is an interesting example of developed expertness. Each veterinary inspector is well grounded in the fundamental knowledge of animal diseases and when entering the service is taken in hand by an experienced inspector for careful instruction in his duties.

In the large packing establishments the post-mortem inspection work is so divided and systematized that each carcass must pass the scrutiny of several inspectors, each of whom gives his entire attention to examining certain particular parts. Under this system the inspector's vision and sense of touch become so highly trained that exceedingly slight variations from the normal in organ or tissue are detected instantly, and the government tag is promptly affixed to all the carcasses which show such variation and they are set aside for further inspection by a final inspector.

---

*Office of Information, U. S. Department of Agriculture, Washington, D. C.

